# The role of phase angle and standardized phase angle in assessing nutritional status and predicting complications in gastrointestinal cancer

**DOI:** 10.1371/journal.pone.0344812

**Published:** 2026-03-13

**Authors:** Dan Zhang, Jiajia Wang, Song Liang, Ping Zhu, Hao Guo, Xinghao Ma

**Affiliations:** 1 Lu’an People’s Hospital of Anhui Province, Lu’an, Anhui, China; 2 Lu’an hospital of Anhui Medical University, Lu’an, Anhui, China; 3 School of Public Health, Anhui Medical University, Hefei, Anhui, China; Fujita Health University: Fujita Ika Daigaku, JAPAN

## Abstract

**Objective:**

This study aims to look at the link between bioelectrical impedance analysis (BIA), especially phase angle (PA) and standardized phase angle (SPA), and how often postoperative infectious complications occur in patients with gastrointestinal tumors.

**Material and methods:**

We chose 139 patients who had gastrointestinal tumor surgery. We did BIA tests on them, assessed their nutritional status with the Patient-Generated Subjective Global Assessment (PG-SGA) scale, checked body composition indicators, and tested their blood markers. The patients were categorized into high PA and low PA groups based on their PA values, and the differences in nutritional status-related indicators and infection rates between these groups were analyzed. Additionally, PA values were standardized, resulting in the formation of high SPA and low SPA groups, and further comparisons of nutritional indicators and infection rates between these groups were conducted.

**Results:**

Our results show that PA and SPA are very important for checking the nutritional status of patients with digestive tract cancer. They are also closely linked to the chance of having postoperative complications. The study encompassed an analysis of 139 patients with digestive tract cancer, leading to several key conclusions: Firstly, a strong correlation was observed between PA and nutritional status, with malnourished patients demonstrating significantly lower PA values compared to those with adequate nutritional status. Secondly, the group with low PA was significantly associated with several adverse indicators, including advanced age, a higher proportion of females, increased prevalence of chronic diseases, lower BMI, elevated PG-SGA scores, higher incidence of sarcopenia, and reduced skeletal muscle mass and skeletal muscle index. Thirdly, patients categorized in the low SPA group exhibited a significantly higher incidence of complications relative to those in the high SPA group. Lastly, high SPA was significantly associated with a lower incidence of postoperative infectious complications, whereas TNM staging was significantly associated with a higher incidence of these complications.

**Conclusion:**

This study substantiates the utility of PA and SPA in evaluating the nutritional status of patients with digestive tract cancer and in being associated with postoperative complications. PA demonstrates a significant correlation with malnutrition, sarcopenia, and various body composition indicators, while SPA is significantly associated with postoperative infectious complications.

## 1 Introduction

Gastrointestinal cancers, including gastric, colorectal, and esophageal cancers—are some of the most common cancers worldwide. They also play a big role in cancer-related illness and death. [1–3]. These cancers are frequently associated with malnutrition, which results from factors such as decreased dietary intake, malabsorption, systemic inflammation, and cancer-associated cachexia [4,5]. Malnutrition is common in these patients. It harms their ability to handle treatment, recovery after surgery, quality of life, and how long they live. [6–9]. This highlights the critical importance of accurate and timely nutritional status assessment in this patient population. Common nutritional assessment tools, like Body Mass Index (BMI), Nutritional Risk Screening (NRS), Mini Nutritional Assessment (MNA), blood markers (albumin and prealbumin), and Subjective Global Assessment (SGA), are widely used in clinics [10–12]. But these methods have built-in weaknesses: they are not good at finding early nutritional problems, and they can be affected by non-nutritional factors like hydration levels or inflammation. As a result, there is an increasing interest in alternative, objective methodologies—such as BIA and metabolomic profiling—that offer a more comprehensive assessment of cellular health and nutritional integrity [13–15]. BIA, in particular, has garnered attention as a non-invasive, rapid, and cost-effective method for evaluating body composition and physiological status. Among the parameters derived from BIA, the PA has emerged as a promising biomarker [15–17]. PA is measured in degrees. It is calculated as the arctangent of reactance divided by resistance, and it shows how healthy cell membranes are. It serves as an indicator of cell membrane integrity, cellular health, and nutritional status. Higher PA values are generally correlated with improved nutritional outcomes, increased muscle mass, and better prognoses in various clinical contexts [18].

Importantly, PA is strongly affected by age, gender, and BMI. This makes it hard to compare PA values across different groups of people. For example, a normal PA for older adults might be too low for younger people. To address this limitation, researchers developed the SPA, which adjusts the measured PA by comparing it to the average PA of a large, healthy reference population stratified by age, gender, and BMI [19]. This adjustment may consequently improve the accuracy of nutritional assessments and the identification of associations with clinical outcomes, particularly in heterogeneous groups such as cancer patients.

Individuals with gastrointestinal cancers frequently experience rapid nutritional decline and elevated rates of complications, including surgical site infections, anastomotic leaks, chemotherapy-induced toxicity, and cachexia-related morbidity [20]. Therefore, there is a pressing need for reliable predictive tools. PA and SPA hold potential as valuable indicators for the early identification of nutritional risk and associations with complications, which may provide insights for personalized interventions, optimize nutritional support, and improve overall care. Despite their potential, PA and SPA have been underexplored specifically in the context of gastrointestinal cancer within recent scientific literature.The majority of existing research primarily addresses general cancer or other diseases, with a paucity of evidence specifically pertaining to gastrointestinal malignancies. This gap underscores the imperative for focused research to substantiate the applicability of these biomarkers within this specific context. The present study seeks to assess the utility of PA and SPA, particularly in their capacity to evaluate nutritional status and predict complications in patients with gastrointestinal cancers. Through a systematic investigation, we aim to elucidate the prognostic relevance (i.e., association with prognosis) of these parameters, facilitate earlier interventions, and ultimately enhance clinical outcomes by improving nutritional management strategies.

## 2 Methods

The study protocol received approval from the Medical Ethics Committee of Lu’an People’s Hospital, Anhui Province (Registration No. 2020LL037). This research is a retrospective analysis, and the data utilized for examination and discussion does not include any individual patient information or sensitive data. Participants were recruited from the Gastrointestinal Surgery Department at Lu’an People’s Hospital in Anhui Province between 01/06/2021 and 31/05/2022. All participants were informed of the purpose and significance of this study, and they signed the informed consent form after giving their consent. The data were collected between 01/06/2021 and 01/07/2023, and were subsequently compiled and analyzed for this study between 20/10/2023 and 12/31/2024. To be included in the study, patients had to be ≥ 18 years old, diagnosed with primary digestive tract tumors (per clinical standards), able to stand on their own, and have no signs of fluid buildup in the chest or abdomen. Exclusion criteria included patients with concurrent tumors at other sites, those who had previously received implantable electronic devices, those who did not undergo PG-SGA evaluation, or those unwilling to participate in body composition analysis using BIA.

Patients who could not complete the PG-SGA assessment after admission were excluded from the study. This decision was made primarily to ensure the reliability and consistency of the assessment data. The primary reasons for non-completion included: (1) clinical instability: such as being in an acute critical condition, having impaired consciousness, or being unable to cooperate due to extreme weakness or pain; (2) cognitive or functional limitations: including severe uncorrected visual or hearing impairment, illiteracy, or cognitive dysfunction due to conditions such as dementia, which prevented understanding or completion of the self-assessment; and (3) patient refusal: a small number of patients declined to participate due to fatigue, discomfort, or the perception that the questions were too intrusive.

This study involved 146 patients diagnosed with gastrointestinal neoplasms who were undergoing surgical treatment. Due to reasons such as lost to follow-up and unwillingness to participate in the study, 7 patients were withdrawn from the study, and ultimately 139 patients were included in the statistical analysis. The nutritional status of these patients was evaluated and monitored using the PG-SGA tool on the preoperative day, while simultaneously measuring PA and SPA values [21]. Of the total participants, 68 were diagnosed with gastric cancer and 71 with colorectal cancer.

### 2.1 Study protocol

Body height was assessed using a measuring tape affixed to the wall. Participants were told to stand with their feet together, heels touching the wall, and keep their bodies straight. The head was positioned in the Frankfurt horizontal plane, ensuring that the lower margin of the orbit and the upper margin of the external auditory meatus were aligned horizontally. The measurer recorded the height by noting the value on the measuring tape at the highest point of the participant’s head. Body weight was determined using a calibrated medical scale. Participants removed heavy outer garments and footwear prior to stepping onto the scale and were instructed to stand still at the center of the platform until the reading stabilized, after which the weight was recorded with precision.

Body composition was evaluated using the Biospace In-Body 770 Body Multi-Frequency Segmental Analyzer [22,23]. This device has a maximum measurement capacity of 300 kilograms and a sensitivity of 0.1 kilograms. This device uses a six-electrode, segmental multi-frequency BIA method. It works at frequencies of 1 kHz, 5 kHz, 50 kHz, 250 kHz, 500 kHz, and 1000 kHz. Participants were required to abstain from eating or drinking for a minimum of 2 hours prior to the measurement. Participants are instructed to remove their coats and socks, standing barefoot with their feet in contact with the foot electrode pads. During the measurement process, the patient grips the palm electrodes with both hands, maintaining their arms at an approximate 45-degree angle relative to the body. The measurement duration is 30 seconds. This device is capable of assessing extracellular water, intracellular water, skeletal muscle mass, body fat mass, body fat percentage, and PA. To ensure the comparability of PA and SPA measurements in patients, minimize measurement errors, and mitigate confounding effects of factors including hydration status, all participants were instructed to fast and refrain from fluid intake for 2 hours prior to testing, and to empty their bladder immediately before the measurement. Measurements were performed between 10:00 a.m. and 12:00 p.m. on the day preceding surgery.

The PG-SGA comprises two components: a self-report by the patient and an evaluation by a trained nutrition specialist. The assessment encompasses seven specific elements: body weight, dietary intake, symptoms affecting food intake, activity and physical function, disease and nutritional requirements, metabolic status, and physical examination. In this study, malnutrition was categorized into three levels: scores of 0–1 indicated a well-nourished status, scores of 2–3 indicated moderate malnutrition, and scores of 4 or above indicated severe malnutrition.

During the patient’s hospitalization, we systematically reviewed their case information to collect data on age, gender, TNM stage, lymph node metastasis, length of stay, postoperative complications, and laboratory test results.

The SPA score was calculated using the formula: SPA = (PA measured – mean PA for age and gender)/ standard deviation of the population for age and gender, as described in previous studies [24,25]. To ascertain the mean and standard deviation of the SPA index, we utilized the values provided by Barbosa-Silva et al [26], which are stratified by gender and age for healthy adults. The accuracy of SPA measurement is influenced by variations in BIA measurements, which result from the absence of unified international standards, differences in manufacturers’ equipment, and ambiguous specifications of electric neutral contact electrodes. Therefore, we selected a cutoff value of-0.4, which is 25% below the SPA value of the study population, to assess the risk of malnutrition [19,23].

### 2.2 Complications

Postoperative infectious complications were defined as infections occurring within 30 days after surgery, including pulmonary infection, abdominal infection (e.g., anastomotic leak-related infection, peritonitis), incision infection (superficial or deep), and infection-induced fever (body temperature ≥38.5°C with positive inflammatory markers or microbiological evidence). All complications were graded using the Clavien-Dindo classification system [27], and only Grade II or higher complications (requiring pharmacological treatment, interventional drainage, or reoperation) were included in the analysis to ensure clinical relevance. Grade II complications included infections managed with systemic antibiotics, Grade III included those requiring interventional procedures (e.g., percutaneous drainage), and Grade IV included life-threatening infections requiring intensive care. This grading system ensures consistency and reproducibility in assessing complication severity [27]. In this investigation, infectious complications were categorized as pulmonary, abdominal, and incision infections, in addition to fever attributable to infection.

### 2.3 Statistical analysis

Statistical analyses were conducted using SPSS version 10.0 (IBM Corp., Armonk, NY, USA). Descriptive statistical methods were utilized to examine the research data. Prior to formal statistical analysis, systematic screening and assessment of missing or incomplete data were performed for all variables. The proportion of missing values for each variable was calculated, and the results showed that the overall missing rate of all variables was < 5%, with no single variable having a missing rate exceeding 10%, so no variable was excluded due to excessive missing values. For handling missing data, targeted strategies were adopted based on variable types and data distribution characteristics. For continuous variables, the Kolmogorov–Smirnov test was used to verify normality [28]. For normally distributed variables with low missing rates, the mean substitution method was employed to impute missing values, which helps maintain the central tendency of the original data. For categorical variables, the mode substitution method was used to fill in missing values, as this is a conservative and effective approach for categorical data with low missing rates. For postoperative follow-up outcome data, missing values were treated as censored data in the survival analysis module of SPSS, and the Kaplan–Meier method was used to account for these censored cases.

For intergroup comparisons of continuous variables, the Independent Sample T-test was applied to data exhibiting a normal distribution, whereas the Mann-Whitney U-test was employed for data not conforming to a normal distribution. The associations between PA, SPA, various indicators, and postoperative infectious complications were explored through univariate logistic regression analysis. Postoperative risk factors associated with complications were identified using multivariate logistic regression analysis. The receiver operating characteristic (ROC) curve was constructed, and the Youden index was computed to determine the optimal PA cut-off value. The results were interpreted within a 95% confidence interval, with statistical significance set at *p* < 0.05.

## 3 Results

### 3.1 ROC curve for the differential diagnosis of malnutrition

This study encompassed a cohort of 139 patients diagnosed with tumors of the digestive tract, comprising 68 individuals with gastric cancer and 71 with colorectal cancer. The cohort included 88 male patients (63.3%) and 51 female patients (36.7%). The PG-SGA scoring revealed that 4 patients were in a good nutritional state, whereas 135 exhibited signs of malnutrition.

We analyzed patients separately based on gastric cancer and colorectal cancer and found that the PA values of 68 gastric cancer patients and 71 colorectal cancer patients were (5.48 ± 0.89)° and (5.16 ± 0.99)°(*p* > 0.05). Upon analyzing their complication outcomes, we found that the complication rates for gastric cancer and colorectal cancer patients were 45.58% and 35.21% (*p* > 0.05). When we split the data by cancer type, we found that in the well-nourished group, gastric cancer patients had a PA of (6.50 ± 0.69)°, and colorectal cancer patients had a PA of (5.80 ± 0.96)° (*p* > 0.05). In contrast, within the malnourished subgroup, the corresponding PA values were (5.29 ± 0.80)° and (5.05 ± 0.95)° for gastric cancer and colorectal cancer patients (*p* > 0.05). These findings collectively indicate that PA exhibits consistent performance in the assessment of gastric cancer and colorectal cancer, regardless of the patients’ nutritional status.

**Fig 1:**
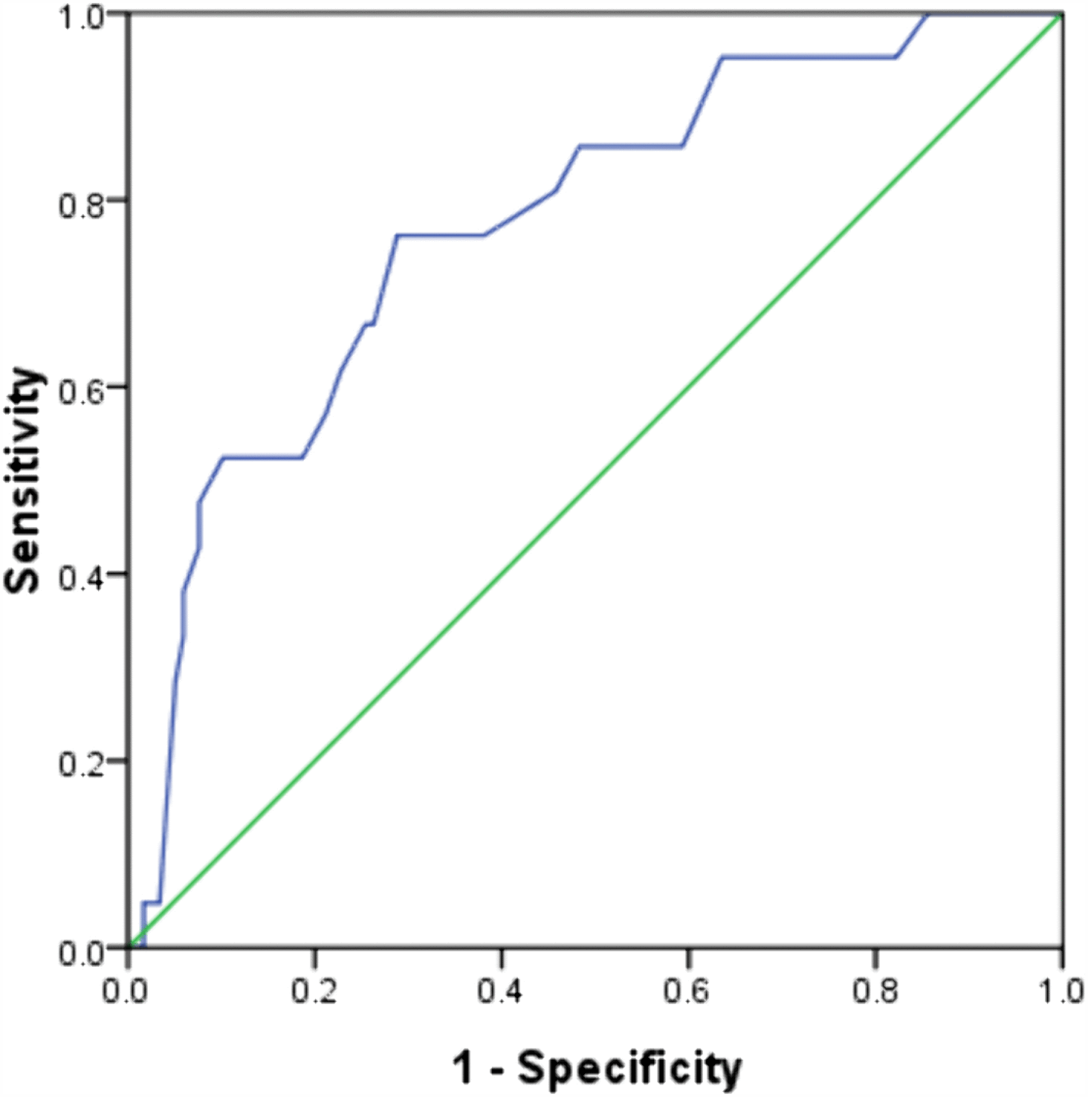
ROC curve for the differential diagnosis of malnutrition.

ROC analysis identified an optimal PA cutoff value of 5.55° for the detection of malnutrition, yielding a sensitivity of 76.2% and a specificity of 71.1%. The model demonstrated substantial discriminatory power, with an area under the curve (AUC) of 77.4% (*p* < 0.001) (**Fig 1**).

### 3.2 Patient characteristics based on PA values

Patients categorized in the low PA group were older, with a median age of 72 years (range 66–78), compared to those in the high PA group, who had a median age of 59 years (range 53–69). Among the 51 female patients, 43 were found to have lower PA levels, representing a significant proportion (84.3%). In male patients, the distribution between the low PA and high PA groups was comparable, with no statistically significant difference observed. However, significant differences were identified in age and gender between the low and high PA groups (*p* < 0.001). The prevalence of chronic diseases was notably higher in the low PA group, at 75.0%, compared to 25% in the high PA group, demonstrating a significant disparity (*p* = 0.013).

The BMI for patients in the low PA group was 22.27 ± 3.65, whereas it was 23.72 ± 3.15 for those in the high PA group, with this difference being statistically significant (*p* = 0.020). According to the PG-SGA scores, only 25.0% of patients in the low PA group exhibited good nutritional status, with moderate and severe malnutrition present in 21.1% and 71.5% of patients, respectively. In contrast, 75.0% of patients in the high PA group had good nutritional status, with moderate and severe malnutrition present in 78.9% and 28.5% of patients, respectively, indicating significant differences between the two groups (*p* < 0.001). The prevalence of sarcopenia was significantly greater in the low PA group, with an incidence of 84.8%, in contrast to only 15.2% in the high PA group, demonstrating a statistically significant difference (*p* = 0.003). Additionally, both skeletal muscle mass and skeletal muscle mass index were significantly lower in the low PA group compared to the high PA group (*p* < 0.001). Conversely, cellular water content and total body water to fat-free mass ratio were higher in the low PA group, with significant differences observed (*p* < 0.001). Blood sample analyses revealed that levels of red blood cells, hemoglobin, albumin, and other relevant indicators were significantly lower in the low PA group than in the high PA group (*p* < 0.05). Although the occurrence of complications did not differ statistically between the two groups (*p* > 0.05), the proportion of patients experiencing complications was higher in the low PA group.

Furthermore, there were no statistically significant differences in tumor location, TNM staging, total fat percentage, or other measured indicators between the two groups (*p* > 0.05). After controlling for gender age and BMI, statistically significant differences persisted in indicators such as the PG-SGA score, BMI, TBW/FFM, and hemoglobin levels (*p* < 0.05) (**Table 1**).

**Table 1:** Patient characteristics based on PA values.

Parameters	All(n = 139)	Low PA group(<5.55^°^) (n = 88)	High PA group(≥5.55^°^) (n = 51)	*p*	Adjusted *p*
**Age**	69 (59-76)	72 (IQR: 66–78)	59 (IQR: 53–69)	<0.001	NA
**Gender, n(%)**				<0.001	NA
**Male**	88 (63.3)	45 (51.1)	43 (48.9)		
**Female**	51 (36.6)	43 (84.3)	8 (15.7)		
**Location, n(%)**				0.154	0.554
**Stomach, n(%)**	68 (48.9)	39 (57.4)	29 (42.6)		
**Intestines, n(%)**	71 (51.1)	49 (69.0)	22 (31.0)		
**Chronic disease, n(%)**				0.013	0.069
**Yes**	60 (43.2)	45 (75.0)	15 (25.0)		
**No**	79 (56.8)	43 (54.4)	36 (45.6)		
**TNM Staging, n(%)**				0.111	0.092
**Ⅰ**	26 (18.7)	12 (46.1)	14 (53.9)		
**Ⅱ**	51 (36.7)	31 (60.8)	20 (39.2)		
**Ⅲ**	48 (34.5)	34 (70.8)	14 (29.2)		
**Ⅳ**	14 (10.1)	11 (78.6)	3 (21.4)		
**BMI**	22.8 ± 3.53	22.27 ± 3.65	23.72 ± 3.15	0.020	NA
**PG-SGA Scores, n (%)**				<0.001	<0.001
**Well-nourished**	4 (2.9)	1 (25.0)	3 (75.0)		
**Moderate malnutrition**	19 (13.7)	4 (21.1)	15 (78.9)		
**Severe malnutrition**	116 (83.4)	83 (71.5)	33 (28.5)		
**Sarcopenia**				0.003	0.137
**Yes**	33 (23.7)	28 (84.8)	5 (15.2)		
**No**	106 (76.3)	60 (56.6)	46 (43.4)		
**Total Fat Percentage**	14.4 (9.0-19.4)	14.4(8.92-19.77)	14.8(9.1-18.5)	0.918	0.255
**Skeletal Muscle Mass**	24.1 (20.7-28.2)	21.9(19.8-26.05)	26.9(25.0-30.3)	<0.001	0.201
**SMI**	7.4 (6.4-8.11)	6.8(6.14-7.7)	8.1(7.5-8.6)	<0.001	0.142
**ECW/TBW**	0.39 (0.38-0.41)	0.40(0.39-0.40)	0.38(0.37-0.39)	<0.001	<0.001
**PA**	5.3 (4.6-6.0)	4.75(4.3-5.2)	6.2(5.9-6.9)	<0.001	NA
**TBW/FFM**	73.7 (73.5-73.9)	73.85(73.7-74.0)	73.5(73.4-73.7)	<0.001	0.748
**Erythrocyte**	4.10 ± 0.6	3.99 ± 0.6	4.34 ± 0.60	0.001	0.086
**Hemoglobin**	118.41 ± 23.8	111.51 ± 22.5	130.26 ± 21.6	<0.001	0.005
**Albumin**	44 (39.2-47.5)	41.6(38.9-46.5)	45.4(41.4-49.6)	0.011	0.059

NA:Adjusted for age, gender and BMI.

### 3.3 Patient characteristics based on SPA values

Utilizing the median SPA value, we established a cutoff of −0.4 to categorize patients into two distinct groups. Patients were classified based on SPA, with those having SPA < −0.4° designated as the low SPA group (n = 70) and those with SPA ≥ −0.4° as the high SPA group (n = 69). The low SPA group had more female patients (66.7% of the group). There was a statistically significant difference in gender between the two groups (*p* < 0.05). Patients diagnosed with intestinal cancer demonstrated lower SPA scores, whereas those with gastric cancer exhibited higher SPA scores, with this difference reaching statistical significance (*p* < 0.05).

Furthermore, the BMI in the high SPA group was significantly greater than that in the low SPA group (*p* < 0.05). In terms of the PG-SGA score, a higher proportion of patients with severe malnutrition was found in the low SPA group (54.3%), whereas 75.0% of patients in the high SPA group were classified as having good nutritional status, with this difference also being statistically significant (*p* < 0.05). The prevalence of sarcopenia was observed to be 78.8% in the low SPA group, compared to only 21.2% in the high SPA group, demonstrating a statistically significant disparity in sarcopenia occurrence between these cohorts (*p* < 0.05). Additionally, both skeletal muscle mass and the skeletal muscle mass index were significantly reduced in patients within the low SPA group relative to those in the high SPA group (*p* < 0.001). Furthermore, individuals in the low PA group exhibited a higher cell water ratio and TBW/FFM than their counterparts in the high PA group, with these differences reaching statistical significance (*p* < 0.001). Blood test analyses revealed that patients in the low SPA group had markedly lower levels of hemoglobin, albumin, and other relevant indicators compared to the high SPA group (*p* < 0.05), although no significant difference was noted in red blood cell counts between the groups (*p* > 0.05). The incidence of complications was significantly greater in the low SPA group, at 65.0%, compared to 35.0% in the high SPA group (*p* < 0.05).

After adjusting for gender and BMI, factors such as tumor location, PG-SGA score, sarcopenia incidence, total fat percentage, skeletal muscle mass, skeletal muscle mass index, TBW/FFM, hemoglobin, and white blood cell count were considered (**Table 2**).

**Table 2:** Patient characteristics based on SPA values.

Parameters	All(n = 139)	Low SPA group (<−0.4^°^) (n = 70)	High SPA group (≥−0.4^°^) (n = 69)	*p*	Adjusted *p*
**Age**	69 (59-76)	69 (59-78)	69 (56.5-73)	0.113	0.225
**Gender, n(%)**				0.003	NA
**Male**	88 (63.3)	36 (40.9)	52 (59.1)		
**Female**	51 (36.7)	34 (66.7)	17 (33.3)		
**Location, n(%)**				0.002	0.008
**Stomach, n(%)**	68 (48.9)	25 (36.8)	43 (63.2)		
**Intestines, n(%)**	71 (51.1)	45 (63.4)	26 (36.6)		
**Chronic disease, n(%)**				0.183	0.072
**Yes**	60 (43.2)	34 (57.6)	25 (42.4)		
**No**	79 (56.8)	36 (46.2)	42 (53.8)		
**TNM Staging (n = 119)**				0.569	0.341
**Ⅰ**	26 (18.7)	10 (38.5)	16 (61.5)		
**Ⅱ**	51 (36.7)	25 (49.0)	26 (51.0)		
**Ⅲ**	48 (34.5)	26 (54.2)	22 (45.8)		
**Ⅳ**	14 (10.1)	8 (57.1)	6 (42.9)		
**BMI**	22.8 ± 3.53	22.04 ± 3.77	23.58 ± 3.11	0.010	NA
**PG-SGA Scores, n(%)**				<0.001	<0.001
**Well-nourished**	4 (2.9)	1 (25.0)	3 (75.0)		
**Moderate malnutrition**	19 (13.7)	4 (21.1)	15 (78.9)		
**Severe malnutrition**	116 (83.4)	63 (54.3)	53 (45.7)		
**Sarcopenia**				<0.001	0.005
**Yes**	33 (23.7)	26 (78.8)	7 (21.2)		
**No**	106 (76.3)	44 (41.5)	62 (58.5)		
**Total Fat Percentage**	14.4(9.0-19.4)	14.4(8.9-18.9)	15.2(9.6-20.0)	0.646	0.001
**Skeletal Muscle Mass**	24.1(20.7-28.2)	22(19.6-25.2)	26.5(23.2-30.1)	<0.001	0.019
**SMI**	7.4(6.4-8.1)	6.75(6.0-7.5)	7.9(7.3-8.45)	<0.001	0.002
**ECW/TBW**	0.39(0.38-0.41)	0.398(0.39-0.40)	0.38(0.38-0.39)	<0.001	<0.001
**PA**	5.3(4.6-6.0)	4.6(4.3-5.0)	6.0(5.3-6.56)	<0.001	<0.001
**TBW/FFM**	73.7(73.5-73.9)	73.85(73.7-74.0)	73.6(73.4-73.8)	<0.001	<0.001
**Erythrocyte**	4.11 ± 0.61	4.02 ± 0.58	4.21 ± 0.64	0.082	0.156
**Hemoglobin**	118.52 ± 23.80	111.36 ± 23.89	125.54 ± 21.73	<0.001	0.002
**Albumin**	44.0 (39.2-47.5)	41.5 (38.3-46.8)	45.1 (41.1-48.2)	0.011	0.021

NA:Adjusted for age, gender and BMI.

### 3.4 Comparison of postoperative complications between the low high SPA group

The overall complication rate was 40.3% (n = 56), with 28.7% (n = 40) identified as infectious complications. As illustrated in **Table 3**, a statistically significant difference in postoperative complications was observed between the low SPA group and the high SPA group (P = 0.019). Notably, significant differences were detected in the incidence of abdominal infection and hypoproteinemia between these groups (*p* < 0.05) (**Table 3**).

**Table 3:** Comparison of postoperative complications between the low SPA group and the high SPA group.

Postoperative Complications	Low SPA group (<−0.4^°^) (n = 70)	High SPA group (>-0.4^°^) (n = 69)	*p*
**Pulmonary Infection**	8 (11.43)	10 (14.49)	0.591
**Abdominal Infection**	9 (12.86)	2 (2.90)	0.030
**Incision Infection**	4 (5.71)	1 (1.45)	0.366
**Fever**	5 (7.14)	1 (1.45)	0.209
**Hypoproteinemia**	6 (8.57)	0 (0.00)	0.028
**The Others**	3 (4.29)	7 (10.14)	0.176
**Overall Complication**	35 (50.00)	21 (30.43)	0.019

### 3.5 Comparison of postoperative outcomes between the low SPA high SPA group

The median hospital stay for patients in the low SPA group was 21 days (IQR 17.0–25.2). This was statistically significantly longer than the high SPA group (*p* = 0.009). The overall hospital mortality rate was 2.1% (n = 3), with no statistically significant differences in 180-day mortality (*p* = 1.00) or 1-year mortality between the two groups (**Table 4**).

**Table 4:** Comparison of postoperative outcomes between the low SPA group and the high SPA group.

Postoperative Outcome	Low SPA group (<−0.4°) (n = 70)	High SPA group (>-0.4°) (n = 69)	*p*
**Total Hospitalization days**	21 (17.0-25.2)	17 (15.5-22.5)	0.009
**Hospital Mortality**	2 (2.86)	1 (1.45)	1.000
**Half-year Mortality**	6 (8.57)	3 (4.35)	0.493
**One-year Mortality**	8 (11.43)	10 (14.49)	0.591

### 3.6 Multiple logistic regression analysis of the impact of postoperative infectious complications

Univariate analysis demonstrated significant associations between age (OR 2.335, 95% CI 1.00–5.429, *p* = 0.049), elevated SPA (OR 0.431, 95% CI 0.201–0.922, *p* = 0.03), and Tumor-Node-Metastasis Classification System (TNM) staging (OR=2.532, 95% CI 1.442–4.458, *p* = 0.001) with the occurrence of infectious complications. In contrast, multivariate analysis identified that TNM staging (OR 5.784, 95% CI 2.288–14.621, *p* < 0.001) was significantly associated with an increased incidence of postoperative infectious complications, whereas high SPA (OR 0.298, 95% CI 0.096–0.922, *p* = 0.036) was significantly associated with a decreased incidence of these complications (**Table 5**).

**Table 5:** Multiple logistic regression analysis of the impact of postoperative infectious complications.

Parameters	B	SE	Wald	*p*	Exp (B)	95%CI
**Age ≥ 60**	1.083	0.592	3.347	0.067	2.954	0.926-9.424
**TNM Staging**	1.755	0.473	13.758	<0.001	5.784	2.288-14.621
**PA(>5.55°)**	0.936	0.682	1.884	0.172	2.551	0.67-9.706
**SPA(<−0.4°)**	−1.211	0.576	4.416	0.036	0.298	0.096-0.922

## 4 Discussion

According to the literature, only 30% to 70% of patients at risk for malnutrition have undergone nutritional assessments, and among those assessed, merely 50% have received nutritional support [19].One potential explanation is that earlier nutritional assessments were often imprecise, highly subjective, and inadequate in accurately reflecting or predicting the prognosis of cancer patients, which contributed to patients’ hesitancy to engage with these assessments [29]. The PA, as an objective indicator of nutritional status, offers a more precise reflection of a patient’s physical condition [30,31]. It minimizes errors associated with subjective evaluations and furnishes clinicians with a more dependable reference point. Utilizing this assessment method enhances the ability to identify associations with patient prognosis and facilitates the development of personalized treatment plans [32].

A major new aspect of this study is using SPA in patients with gastrointestinal cancer. This fixes a big problem with the usual PA. PA is a well-known objective measure of cellular health, but its use is limited by age, gender, and BMI. These factors vary widely among cancer patients (e.g., a “normal” PA for older adults may be abnormally low for younger individuals) [33]. By standardizing measured PA against age-, gender-, and BMI-matched healthy reference values, SPA eliminates demographic-related variability and provides a more intrinsic assessment of nutritional and cellular integrity.

This study corroborates a significant association between PA and the nutritional status of patients with digestive tract cancer. PA serves as an indicator of cell membrane integrity and cellular functional status. It is important to emphasize that PA and SPA are complementary to, rather than replacements for, established nutritional assessment tools such as the PG-SGA. The PG-SGA remains a well-validated gold standard for nutritional assessment in cancer patients, as it comprehensively integrates subjective patient-reported data (e.g., dietary intake, symptom burden) and objective clinical evaluations (e.g., physical examination, disease severity) to capture the holistic nutritional status [21]. In contrast, PA and SPA provide unique objective insights at the cellular and tissue levels, specifically reflecting cell membrane integrity, skeletal muscle quality, and hydration balance, that are not fully captured by the PG-SGA or traditional anthropometric/biochemical indicators [26,31]. For example, PA may detect early cellular-level nutritional deficits in patients with normal PG-SGA scores (e.g., subtle sarcopenia or impaired membrane function), while the PG-SGA can contextualize these BIA-derived parameters by identifying underlying causes (e.g., appetite loss, malabsorption). Together, the integration of PA/SPA with the PG-SGA enhances the comprehensiveness and accuracy of nutritional assessment.

Malnutrition is frequently accompanied by compromised cell membrane integrity and impaired cellular function, resulting in reduced PA values [34–36]. In this investigation, the PA of malnourished patients was significantly lower than that of well-nourished patients, aligning with the findings of Sandini et al [37]. Furthermore, PA is closely linked to sarcopenia.

The link between low SPA and more postoperative infectious complications may come from two connected reasons. SPA may directly reflects skeletal muscle quality and cellular membrane integrity key determinants of immune cell function and tissue repair capacity. Poor cellular health (shown by low SPA) reduces the body’s ability to fight infections and heal surgical wounds. Similarly, we also believe that low SPA correlates with reduced hemoglobin and albumin levels, which further exacerbate infection risk by impairing oxygen transport to tissues and reducing the synthesis of immune-active proteins.

The prevalence of sarcopenia was significantly higher in the low PA group compared to the high PA group, mirroring findings from studies on the Korean population [38]. Additionally, this study observed that patients in the low PA group exhibited poorer body composition indicators, including reduced SMM and SMI, as well as an elevated TBW/FFM ratio. These observations are consistent with the fundamental principle of bioelectrical impedance: a decrease in cell mass and an increase in extracellular water lead to reduced electrical impedance and PA [30,39]. Regarding hematological indicators, the concentrations of red blood cells, hemoglobin, and albumin were markedly reduced in the low PA group, thereby reinforcing the association between PA and overall nutritional status [40,41].

A novel aspect of this study is the introduction of the SPA concept, which has demonstrated a significant association with postoperative complications, particularly infectious ones. SPA, defined as the standardized value of the PA in relation to individual expected values, considers variables such as age, gender, and BMI. This approach potentially offers a more precise assessment of an individual’s nutritional and cellular health status compared to the traditional PA.

The study revealed that the overall complication rate was significantly higher in the low SPA group compared to the high SPA group, with a high SPA being significantly associated with a lower incidence of complications. These findings align with the research conducted by Sandini et al. in Italy [37]. The association of SPA with infectious complications may be attributed to its ability to reflect cellular immune function and tissue repair capacity. Moreover, a low SPA is correlated with reduced hemoglobin and albumin levels, both recognized as risk factors for infection. This study underscores the utility of PA and SPA as significant tools for nutritional evaluation and the identification of associations with complications in patients with digestive tract cancer. PA offers cellular-level insights that surpass those provided by traditional anthropometric measurements or biochemical indicators.

In clinical settings, PA can serve as a screening mechanism for identifying high-risk patients. Existing research has demonstrated that PA is an effective screening instrument for detecting malnutrition [42,43], sarcopenia, and complications in gastric cancer patients, aligning with our findings. The incorporation of SPA may enhance predictive accuracy by considering individual characteristics such as age, gender, and BMI. Additionally, PA and SPA can be employed to monitor the efficacy of interventions. For example, an increase in PA following nutritional support or physical exercise may reflect an improvement in cellular health. Although this study did not include interventions, the potential application of this approach warrants further investigation.

The evaluation of nutritional status, development of individualized nutritional plans, provision of suitable nutritional support, and implementation of enteral nutrition programs for all patients are consistently overseen and continuously monitored by a single, experienced dietitian throughout the entire process. A notable aspect of this study is the completion of BIA measurements by the same dietitian.

Notably, this study has inherent limitations that should be considered when interpreting the findings: the single-center retrospective design, small sample size, and lack of interventional validation limit the generalizability and causal inference of our results. Residual confounding from perioperative factors (e.g., neoadjuvant therapy, intraoperative fluid management) may also have influenced outcomes. These limitations highlight critical directions for future research that directly link to clinical translation: 1) Validate the SPA cutoff value in large-scale, multi-center cohorts to establish standardized clinical thresholds for gastrointestinal cancer patients; 2) Develop SPA guided personalized nutritional intervention protocols (e.g., tailored protein supplementation, resistance training) and prospectively evaluate their efficacy in reducing postoperative infectious complications; 3) Integrate SPA into routine preoperative risk stratification tools to identify high-risk patients early and optimize perioperative management (e.g., prolonged nutritional support before surgery). Additionally, exploring the longitudinal changes of SPA during cancer treatment and its association with long-term survival may further expand its clinical utility.

## 5 Conclusion

This study substantiates the utility of PA and SPA in evaluating the nutritional status of patients with digestive tract cancer and in being associated with postoperative complications. PA demonstrates a significant correlation with malnutrition, sarcopenia, and various body composition indicators, while SPA is significantly associated with postoperative infectious complications.

## Supporting information

S1 Table The raw data of the study. (XLSX)
